# Untangling the Relationship Between Internalized Heterosexism and Psychological Intimate Partner Violence Perpetration: A Comparative Study of Lesbians and Bisexual Women in Turkey and Denmark*

**DOI:** 10.1177/08862605211004108

**Published:** 2021-04-20

**Authors:** Esra Ummak, Ezgi Toplu-Demirtaş, Reidar Schei Jessen

**Affiliations:** 1 University of Oslo, Norway; 2 MEF University, Istanbul, Turkey; 3 Florida State University, Florida, USA; 4 Oslo University Hospital, Oslo, Norway

**Keywords:** lesbian and bisexual women, psychological intimate partner violence perpetration, internalized heterosexism, cross-cultural research intersectionality, minority stress

## Abstract

Psychological intimate partner violence (IPV) perpetration is not limited to heterosexual relationships and can affect all genders and sexual orientations, including lesbians and bisexual women (LB) both in Denmark and Turkey. Internalized heterosexism might be one of the factors increasing the risk of LB’s use of psychological IPV perpetration. However, it is still unclear how being LB in Turkey and Denmark interact in the internalized heterosexism and psychological IPV perpetration relationship. The current study, therefore, presents an investigation of (a) the prevalence of sexual orientation (LB) and country (Denmark and Turkey) differences in perpetrating psychological IPV and (b) the moderating roles of sexual orientation and country on the association between internalized heterosexism and psychological IPV perpetration. A sample of 449 LB from Denmark and Turkey completed the Lesbian Internalized Homophobia Scale and the Multidimensional Measure of Emotional Abuse Scale. The results of chi-square analyses indicated that LB from Turkey and bisexual women from both countries reported significantly higher psychological IPV perpetration. The results of moderation analyses revealed that country had direct effects on the use of psychological IPV perpetration. No moderation effects were found for both sexual orientation and country in three of the four types of psychological IPV perpetration. These findings suggest that LB are not an exception to the perpetration of IPV. Furthermore, the findings were discussed from the perspectives of intersectionality and minority stress.

The work presented here was largely performed while the first author was a post-doctoral researcher at Copenhagen University [and is now at Oslo University].

While the mainstream approach to understanding intimate partner violence (IPV) has been dominated by heteronormativity and gender binary conceptualization, IPV is not specific to heterosexual relationships. Some studies have found similar prevalence rates of IPV in heterosexual relationships and lesbian, gay, and bisexual (LGB) relationships (Gonzalez-Guarda et al., 2013), while other studies suggest that IPV is significantly more prevalent among LGBs than heterosexuals (Blosnich & Bossarte, 2009; Dank et al., 2014; Reuter et al., 2015). According to Meyer (1995, 2003), the minority stress model indicates that contextual triggers for IPV, together with internalized heterosexism and an expectation to be discriminated against, are the same predictors as for higher mental health challenges among LGB people. Therefore, we suggest that IPV among LGB people should be analyzed using the minority stress model to shed light on potential mediators between internalized heterosexism and IPV. From an intersectional perspective, the LGB community faces unique challenges concerning IPV and contributing factors because of their sexual and gender minority status. To adequately understand the dynamics of IPV among lesbian, gay, bisexual, transgender, and queer (LGBTQ) people, researchers need to reframe the existing gender-based conceptualization through an intersectional perspective within the minority stress model. Accordingly, in this study, we focused specifically on lesbian and bisexual women (LB).

## Prevalence of Psychological IPV Perpetration among LB

In a meta-analysis of IPV among lesbians, the lifetime prevalence of violence perpetration was 27% (Badenes-Ribera et al., 2015). In another study, Matte and Lafontaine (2011) reported that the rates of psychological abuse perpetration were 76.2% in a sample of 143 women (77.6% identified as lesbian and 22.4% identified as bisexual). Studies on differences in psychological violence perpetration between LB are very scarce, and prior research on IPV has generally focused on lesbians. Balsam and Szymanski (2005) reported that more bisexual women (46.2%), compared to lesbians (15.2%) were likely to report LGB-specific tactics of psychological aggression (e.g., “I forced my partner to show physical or sexual affection in public, even though she didn´t want to”) against a female partner in the past year.

In Denmark, there is limited data on the prevalence of IPV, and no scientific study has examined IPV specifically among the LB population. A survey conducted among the 28 member states of the European Union (EU) by the European Union Agency for Fundamental Rights (FRA, 2014) found that the lifetime prevalence of psychological violence against women by intimate partners was among the highest in Nordic countries of the EU (FRA, 2014). In particular, Denmark had the highest prevalence with 60%, followed by Finland with 53%, and Sweden with 51%.

In Turkey, whilst IPV has been studied more extensively, only one report examines IPV among LB. Ayhan-Balik and Bilgin (2019) found that all forms of IPV perpetration occurred among lesbians in Turkey, but the most prevalent type was psychological violence (66.4%). Keeping the limited studies on psychological IPV among LB in mind, our first aim in the current study was to investigate the prevalence rates of psychological IPV perpetration among LB in relation to sexual orientation and country.

### The Minority Stress Model

Health disparities among LGBTQ+ people are commonly associated with social and contextual adversities due to continued discrimination and lack of protection against discrimination. More specifically, the LGB population demonstrates a higher prevalence of mental health challenges than heterosexuals (Meyer, 2003). The theory of minority stress is a conceptual framework developed to understand the excess of mental health challenges among gender and sexuality minorities (Meyer, 1995, 2003). The minority stress theory suggests that stigma, prejudice, and stressful social environments cause mental health problems, and has been found useful for studying the associations between mental health problems and psychosocial challenges such as alcohol abuse and perpetration of violence, and gender and sexuality minorities, as well as to develop strategies for improving well-being (see, for example, Chodzen et al., 2019).

Minority stress refers to the distress experienced by individuals from stigmatized social groups due to their minority position (Meyer, 2003). Therefore, minority stress is assumed to be an additive to general stressors because it is chronic, related to stable underlying social and cultural structures, and socially based. Previous research has indicated that heteronormativity and heterosexism can lead to the perpetration of violence (Balsam, 2001; Tigert, 2001) in LB romantic relationships. For instance, Tigert (2001) argues that the perpetration of violence might be a response to cultural oppression/traumatization.

The minority stress theory posits that three factors contribute to the distress experienced by the LGB population: (a) external, objective, and stressful events and conditions, such as a lack of institutional support (i.e., marriage); (b) expectations of such events and the associated vigilance; and (c) internalization of negative social attitudes, referred to here as internalized heterosexism (Meyer, 1995). Although the term internalized homophobia is more frequently used in the literature, in this paper we prefer to use internalized heterosexism due to the emphasis on social and cultural issues rather than personal ones (Herek, 1995). Moreover, unlike phobias, homophobia is underpinned by heteropatriarchal values and male dominance beyond irrational fear (Pharr, 1988; Rich, 1980). Besides, the concealment of sexual orientation is suggested as a distinct stressor for the LGB population (Meyer, 2003).

One example of a stress-ameliorating factor is a sense of belonging to a cohesive social group that enables a favorable comparison of one’s own self and identity with others (Meyer, 2003). Within this framework, the individual minority member’s unique self-identity is, of course, a mediating mechanism of the effect of minority stress. Individuals with more complex identities that are not deeply fused with a minority position, but integrated with other aspects of the person’s life, are more resilient to minority stress. However, individuals who do not identify with a minority position might be more prone to minority stress because of a lack of group solidarity associated with group belongingness. This could be because whilst a person does not identify as (for example) a lesbian, they may be labeled and treated by others as a member of the stigmatized minority, because of having a same-sex partner (Meyer, 2003).

### Internalized Heterosexism and Psychological IPV

Various mechanisms have been proposed to explain the relationship between internalized heterosexism and IPV. Balsam (2001) suggests that internalized heterosexism might restrain the connection with LGB communities, and this isolation can create a sense of dependency on the partner; in other words, a sense of “fusion.” Renzetti (1992) found that the perpetrator’s dependency on their partner leads to conflict and severe violence. Moreover, Balsam and Szymanski (2005) stated that women who have internalized negative cultural beliefs and attitudes about being LB might feel negatively about themselves, and this may lead them to believe that they “deserve” the abuse perpetrated in that relationship. Thus, the perpetrator indirectly uses her partner’s internalized heterosexism to justify using violence. Therefore, the differences in internalizing heterosexism might affect the prevalence rates of psychological IPV perpetration among LB (Balsam & Szymanski, 2005; Gulmez, 2018; Lewis et al., 2014; Matte & Lafontaine, 2011).

### Intersectionality

To be more precise when studying potential moderators (in a psychological context), an intersectional framework has been proposed (McCormick-Huhn et al., 2019). At its core, the concept of intersectionality emphasizes that we cannot understand the individual’s experience of one social group membership without taking into account other social group memberships (Crenshaw, 1989). For example, when studying the experiences of women, it is crucial to acknowledge that Black women might be affected differently than White women. Crenshaw (1989) has devoted her research to the experiences of women of color. However, she states explicitly that other social group memberships, such as being lesbian or bisexual, should also be considered when studying the risk of marginalization among women. Thus, if one aims to identify preventive measures in order to decrease the incidence of IPV among LB, it is pivotal to take into consideration the potentially unique experiences of sexual minority women in comparison to heterosexual women. Furthermore, when analyzing a social group such as LB, one should take other social backgrounds among LB into consideration, for example, class, education, and ethnicity. Besides, an essential aim of the intersectional perspective is to highlight how power relationships are reflected between social groups. More specifically, McCormick-Huhn et al. (2019) suggest four insights from the intersectional perspective that can help psychologists to recognize how the different minority backgrounds of research participants, for example, being lesbian or bisexual, might affect the results and analysis of findings: (a) participants are multidimensional and might belong to different social groups; (b) participants’ social group memberships are not stable and fixed, but dynamic across time and place; thus, people can be strategic in their self-presentation, and it is important to acknowledge historical and geographical contexts; (c) participants’ intersectional positions regulate access to power; and (d) participants’ intersectional positions might affect the results and generate different causal mechanisms.

From an intersectional perspective, lesbian and bisexual women are uniquely different despite the similarities between sexual orientations due to their gender and minority status (Szymanski & Owens, 2008). For example, bisexual women have to cope with internalized bi-negativity as well as internalized heterosexism due to the normative sexual dichotomy of society (Guidry, 1999). At this point, there might be differences between LB regarding psychological violence perpetration. Balsam and Szymanski (2005) found that although lesbians reported more lifetime psychological aggression, bisexual women reported more recent LGB-specific psychological aggression.

### Why Turkey and Denmark?

From an intersectional perspective, reflection on the effects of cultural, national, and regional frames is important when studying IPV among LGB people, especially how this intersects with the unique experiences of being lesbian or bisexual (Baker et al., 2013; Burke et al., 2002). To study the potentially different causal mechanisms underlying experiences of stress and stigma, as outlined by McCormick-Huhn et al. (2019) in their four insight, one has to collect data from research participants that belong to the same social category membership but share different experiences related to other aspects of their background, for example, nationality, culture, and social class. This makes Turkey and Denmark relevant because of the social, cultural, and political differences between the two countries, especially related to the living conditions of the LGBTQ+ populations. Still, we know that IPV perpetration among LB occurs in both countries. Studies that compare the prevalence of IPV perpetration and internalized heterosexism across countries with different living conditions are ideal for an intersectional analysis because it allows comparison between the same social group living in countries with varying conditions of living (McCormick-Huhn et al., 2019). In the following, we will describe the most important parameters that make Turkey and Denmark different, more precisely the individualism-collectivism-axis, the legal situation, and the local social group movements for LB. Thus, from an intersectional perspective, potential divergent results between LB in Turkey and Denmark could be related to national differences (McCormick-Huhn et al., 2019).

According to Hofstede’s cultural dimensions theory (Hofstede, 2011), Turkey scores 37 and Denmark 74 on the individualism dimension (Hofstede Insights, n.d.). This indicates that Turkish society has more collectivist tendencies; for example, family needs and values are more dominant than individual needs when compared to Danish society, which primarily values individualism. Using the same theory (Hofstede, 2011), Turkey scores 85 and Denmark 23 with regard to the uncertainty avoidance dimension (Hofstede Insights, n.d.), indicating stronger uncertainty avoidance tendencies among Turkish society compared with Danish society; for example, being intolerant of deviant persons and ideas, (in other words, what is different is more dangerous). It has been indicated generational differences regarding attitudes toward the LGB population in Turkey (Oksal, 2008). The parent generation expressed in one study relatively negative attitudes toward LGB individuals. In the younger generation, the picture was a bit more complicated; men express relatively negative attitudes toward both lesbians and gays. Young women, on the other hand, are relatively negative toward lesbians, but more positive toward gays. On the other hand, the family model is legally inclusive of LGBTQ+ people since 2012 in Denmark. However, in Turkey, the family model is still heteronormative and heterosexist and exclusive of LBGTQ+ people (Zengin, 2019). A cross-national study of psychological IPV among LB in Turkey and Denmark might help understand the potential effect of culture on IPV, utilizing data collected from a collectivistic country and an individualistic country.

Legal regulations and practices regarding the rights of LGBTQ+ people in Denmark and Turkey are quite different. Using the current Rainbow Index published by the European Region of the International Lesbian, Gay, Bisexual, Trans, and Intersex Association (ILGA-Europe, 2020), Denmark ranks 4th (68% on a scale of 0-100), whereas Turkey ranks 48th (4%) in terms of respect for human rights for LGBTQ+ individuals. Moreover, according to the ILGA-Europe (2019), a ban on public events organized by LGBTQ+ groups, a lack of hate crime or hate speech laws, and widespread discrimination towards LGBTQ+ people still exist in Turkey. Conversely, in Denmark the government established a minister to coordinate LGBTQ+ rights and nondiscrimination and announced Denmark´s first-ever LGBTQ+ action plan. Furthermore, LGBTQ+ nongovernmental organizations in Denmark received 1.5 million DKK core funding from the state, and Sabaah, which is an LGBTQ+ organization working with ethnic minorities, obtained funding of 2.9 million DKK as part of the action plan. In Turkey, there is no protection against LGBTQ+ discrimination, whereas in Denmark, LGBTQ+ discrimination has been illegal since 1996, and LGBTQ+ employment discrimination was prohibited from 2017 (Equaldex, 2019).

The LGBTQ+ political and social movement in Turkey became visible in the late 1980s, particularly in the 1990s, thanks to the formation of associations such as Lambda Istanbul in İstanbul and Kaos GL in Ankara. In the 2000s, many more organizations (such as independent initiatives, associations, and university student clubs) emerged in the larger cities (Özkan, 2004). In other words, small cities remain excluded from these movements. In addition, in the past two years, the Ankara Governorship has banned activities of LGBTQ+ organizations such as cinemas, theaters, panels, interviews, and exhibitions. Istanbul Pride was also banned in 2015, having taken place for the previous 27 years (ILGA-Europe, 2019). By contrast, the LGBTQ+ movement in Denmark started in 1948. The biggest and oldest LGBTQ+ organization “LGBT Denmark” was founded in 1948 under the name Kredsen of 1948 (Circle of 1948) by a circle of friends of the activist Axel Lundahl-Madsen—later Axgil (Edelberg, 2015)—and Lesbisk Bevægelse–LB (Lesbian Movement) founded by activist lesbians in 1974. Lesbian weeks have been organized at a camp on the appropriately named island of Femø since 1974, and Pride has been held in Copenhagen since 1996.

Despite the increasing improvements in legal regulations and practices in Denmark, research has indicated that LGBTQ+ individuals both in Denmark (Hansen & Gransell, 2009) and Turkey (Baydar, 2015) continue to experience mental health problems, such as suicidal thoughts and depression. Even now, the normalizing hegemony of heterosexuality may still be placing LGBTQ+ individuals in jeopardy in both countries, even though significant differences exist between them concerning legal and societal acceptance of nonheterosexuality.

By comparing individuals from the same social group membership living in different countries, one could increase knowledge and understanding of the various intersections between stigma and social background that contribute to IPV among LB. From an intersectional perspective, a comparison of the relation between IPV perpetration and internalized heterosexism among LB in Turkey and Denmark could offer an insight into the potential role of legal protection regarding sexual minorities, different societal attitudes toward LGBTQ+ people, and cultural differences related to individualism and collectivism in developing and preventing IPV among LB. Furthermore, increased knowledge on the relation between IPV perpetration and internalized heterosexism could help us learn more about whether and how social attitudes toward sexual minority groups and legal and social recognition influence the development of sexual gender identity among LB living in different countries.

### The Current Study

In this study, we first aimed to explore the frequency of psychological IPV perpetration through four distinct forms of aggression—restrictive engulfment, denigration, hostile withdrawal, and dominance/intimidation—with a focus on differences according to potential sexual orientation and country. We decided to focus on psychological violence, since this is estimated to be the most common form of IPV in Europe and the USA. Furthermore, despite the significant prevalence, the documentation of the effects of psychological IPV on mental health and the understanding of the underlying mechanisms are scarce (Dokkedahl et al., 2019). Thus, to prevent IPV, more knowledge is needed on the effects of psychological IPV and the factors that contribute to it (Dokkedahl et al., 2019). Additionally, psychological violence is not perceived as “serious and harmful” as physical violence (Donovan & Hester, 2015). Thus, we believe that it is crucial to make psychological violence and its adverse effects on LB’s mental health visible, keeping the intersectionality of sexism, heterosexism, and internalized heterosexism in mind.

By psychological IPV, we refer to “coercive and aversive acts intended to produce emotional harm or threat or harm and directed at target's emotional wellbeing or sense of self” (Murphy & Hoover, 1999, p. 40). To measure the concept, we used the Multidimensional Measure of Emotional Abuse (MMEA; Murphy & Hoover, 1999), which enabled us to explore psychological aggression in great detail because of its multidimensionality and some other characteristics (please see Toplu-Demirtaş et al., 2018 for further characteristics). Due to the scarce body of knowledge on the relation between LGBTQ individuals and IPV, we decided to focus exclusively on IPV perpetration in the present study in order to investigate the characteristics and the perspective of the perpetrator in more detail. This was also done because the literature on minority stress has focused on the relationship between stigma, discrimination and psychosocial stressors such as alcohol abuse and perpetration of violence in close relations (Meyer, 2003). The binary identities of subjects of IPV perpetrators in heterosexual relationships (men as perpetrators and women as victims) seem to be both misleading and unprecise in the contexts of LGBTQ+ because members of this population tend to divert from heteronormative expectations regarding behavior and gender identity. Moreover, the male as perpetrator dominance in the violence literature leaves little room to discuss lesbian battering. For the very point, we believe investigating violence in the context of “women and perpetrator” is both crucial and will contribute to the literature.

Regarding our first purpose, we hypothesized the following:

Because of the reflection of societal and institutional discrimination on women in daily practices, LB in Turkey will use more psychological violence toward their partners than LB in Denmark.

Because of double discrimination, from both heterosexual and lesbian communities, bisexual women will use more psychological violence toward their partners than lesbians, regardless of the culture.

**Figure 1.**
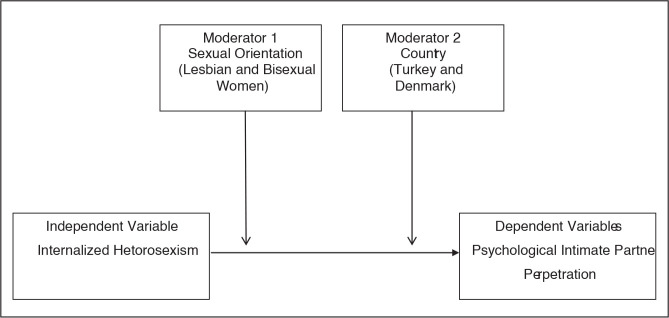
Sexual Orientation and Country as Moderators of the Association Between Internalized Heterosexism and Psychological Intimate Partner Perpetration^1^.

*Note.* We have four dependent variables of psychological intimate partner perpetration; restrictive engulfment, denigration, hostile withdrawal, and dominance/intimidation.

For our second aim, we investigated the moderating roles of sexual orientation (specifically LB) and country (Turkey and Denmark) on the association between internalized heterosexism and the use of psychological IPV (please see Figure 1). Based on prior research and with our second purpose in mind, we hypothesized the following:

There will be a positive association between internalized heterosexism and psychological IPV perpetration.

The country will moderate the association between internalized heterosexism and psychological IPV perpetration.

Sexual orientation will moderate the association between internalized heterosexism and psychological IPV perpetration.

## Method

### Procedure

Following ethical approval from Copenhagen University (the Institutional Ethical Review Board), data were collected through a purposeful sampling procedure via an online survey. While the ethical approval was granted from a Danish university [Copenhagen University], the Turkish survey followed the same ethical standards, and it was indicated in the consent form. The scales were administered based on voluntary participation, and informed consent was provided from each participant. The survey included a warning indicating that some questions may trigger emotional responses and encouraging participants to consider their mental well-being before participating in the study. Nearly 80 different LGBTQ+ organizations, and queer and feminist groups in both Denmark and Turkey were contacted to introduce the study to members. Most LGBTQ+ nongovernmental organizations shared the link for the survey on their social media accounts, and some sent the survey to their group members, with a reminder once a month for four months. We endeavored a lot (surveying online through communicating with nearly 80 different LGBTQ+ organizations, queer and feminist groups, and NGOs in Turkey and Denmark with subsequent reminders one month apart for four months) to reach and ensure the representative cohorts of participants. The participants were thanked and informed about the study after they completed the scales. The completion of the questionnaire took about 10-15 minutes. We followed the same procedures for the distribution of the survey in both countries. We could not estimate the response rate since Google Forms provides access only to completed data and does not show how many potential respondents received and not finished the survey.

### Participants

In total, 536 individuals from Turkey and Denmark participated in this study. The participants were selected based on four inclusion criteria: (a) voluntary participation, (b) aged over 18, (c) gender identity defined as a woman and sexual orientation defined as lesbian or bisexual, and (d) having a current or previous romantic relationship (dating, cohabiting, engaged, or married). Of 536 participants, 9 (1.70%) were excluded as they had never been involved in a romantic relationship. Because we were interested in people who specifically identified as lesbian and bisexual women, we further removed 12 cases from Turkey and 66 cases from Denmark who identified as heterosexual and other.

Of the 449 remaining participants, 271 were from Denmark (60.40%), and 178 were from Turkey (39.60%). As presented in Table 1, participants from Denmark and Turkey were aged between 17^1^ and 71 years (*M* = 32.91, *SD* = 11.09) and between 18 and 53 years (*M* = 29.06, *SD* = 7.81), respectively. The average relationship length was 54.52 months for participants in Denmark (*SD* = 64.58; *min* = 1 and *max* = 384 months) and 32.42 months for those in Turkey (*SD* = 34.05; *min* = 1 and *max* = 204 months). One apparent difference between both countries was the statement on sexual orientation. More women in Turkey identified themselves as bisexual (57.90%) rather than lesbian (42.10%), compared with women in Denmark, of whom 62.40% were lesbian and 37.60% bisexual. Considering relationship status, more participants in Denmark defined their relationships as married (18.50%) than those in Turkey (6.20%).

**Table 1.** Demographic Characteristics of Participants in Denmark and Turkey.

	Denmark(*N* = 271; 60.4%)	Turkey(*N*= 178; 39.6%)
**Gender identity**		
Cis-gender	252 (93.30%)	166 (93.30%)
Trans-gender	10 (3.70%)	9 (5.10%)
Complex	9 (3.35%)	3 (1.70%)
**Sexual orientation**		
Lesbian	169 (62.40%)	75 (42.10%)
Bisexual	102 (37.60%)	103 (57.90%)
**Relationship status**		
Current	202 (74.50%)	123(69.10%)
Previous	69 (25.50%)	55 (30.90%)
**Type of relationship**		
Dating	63 (23.20%)	47 (26.40%)
Cohabiting	55 (20.30%)	36 (20.20%)
Long-distance	18 (6.60%)	26 (14.60%)
Engaged	8 (3.00%)	2(1.10%)
Married	50 (18.50%)	10 (6.20%)
Other	4 (1.50%)	11(1.50%)
No current	73 (26.90%)	46 (25.80%)
**Perception of relationship**		
Stable/serious	178 (69.30%)	92 (60.50%)
Casual	8 (3.10%)	2 (1.30%)
Don’t know/uncertain	4 (1.60%)	14 (9.20%)
Other	2 (.080%)	10 (6.60%)
No current	65 (25.30%)	34 (22.40%)
Age (M + SD)	32.91 (11.09)	29.06 (7.81)
Relationship length	54.52 (64.58)	32.42 (34.05)

*Note.* Complex. My gender identity status is more complicated. I am neither cis nor trans.

### Data Collection Instruments

#### Demographics

We collected demographic data, including sexual orientation, gender identity, age, and relationship characteristics via a survey form we created.

#### Internalized Heterosexism

We measured internalized heterosexism using the modified version of the Lesbian Internalized Homophobia Scale (LIHS; Szymanski & Chung, 2008). The LIHS is comprised of 52 items across 5 factors: connection with the lesbian community (e.g., “Most of my friends are lesbians, bisexuals, or queer”), public identification as a lesbian (e.g., “I would not mind if my boss knew that I was a lesbian, bisexual, or queer”), personal feelings about being lesbian (e.g., “I am proud to be a lesbian, bisexual, or queer”), moral and religious attitudes towards lesbians (e.g., “Lesbian, bisexual, and queer couples should be allowed to adopt children the same as heterosexual couples”), and attitudes towards other lesbians (e.g., “Lesbians, bisexuals, and queers are too aggressive”). The LIHS is rated on a 7-point Likert-type scale (1 = *very strongly disagree* to 7 = *very strongly agree*) with higher scores reflecting more internalized heterosexism. The evidence on validity and reliability for the LIHS was provided by Ozturk and Kindap (2011). For the Danish version, the scale was utilized in English, and the results of the preliminary analysis on its validity and reliability were satisfactory.

We calculated the total score of the LIHS based on the sum of the 52 item scores. In our study, Cronbach’s alpha was .91, and .90 for the samples from Denmark and Turkey, respectively.

#### Psychological IPV Perpetration

To assess psychological IPV perpetration, we used the MMEA Scale (Murphy & Hoover, 1999). This inventory has four subscales, as follows: (a) restrictive engulfment (including 7 items, e.g., “I secretly searched through my partner’s belongings”); (b) denigration (including 7 items, e.g., “I called my partner worthless”); (c) hostile withdrawal (including 7 items, e.g., “I acted cold or distant when angry”); and (d) dominance/intimidation (including 7 items, e.g., “I threw, smashed, hit, or kicked something in front of my partner”). Participants’ responses were collected on a 7-point frequency scale (from 0 = *never* to 6 = *more than 20 times*) for a period of 6 months prior to taking part in the study. A total score was calculated by summing the item responses for each subscale, such that higher scores reflected more frequent use of the psychological form of IPV. The validity and reliability evidence of the MMEA Scale in Turkish was proven by Toplu-Demirtaş et al. (2018). For the Danish population, the English version was used, and preliminary analysis on its validity and reliability was satisfactory.

In this study, Cronbach’s alpha was .73 for restrictive engulfment, .71 for denigration, .87 for hostile withdrawal, and .85 for dominance/intimidation for the Danish sample, whilst for the Turkish sample, the Cronbach’s alphas for the four subscales were .78, .79, .84, and .83, respectively.

### Data Analysis

We used frequency and chi-square analyses to explore the frequencies of IPV perpetration and to determine whether differences emerged concerning sexual orientation and country, respectively (see Table 2). Then, Pearson correlations were utilized to examine associations among study variables (see Table 3). To test the hypothesized moderation model, we conducted a moderation analysis using Hayes’ (2019) PROCESS (Version 3.4, Model 2). Bootstrapping was used to handle non-normality and test direct and indirect effects (Preacher & Hayes, 2008).

## Results

### Prevalence Rates of Psychological IPV Perpetration

We first investigated prevalence rates of psychological IPV perpetration. For this reason, we dichotomized the composite of psychological violence types into 0 (never perpetrated an instance of abuse in the past 6 months) and 1 (perpetrated at least one instance of abuse in the past 6 months) for previous and current relationships of LB. With regard to country, LB in Turkey reported using significantly more psychologically aggressive behaviors of all types toward their partners, as presented in Table 2. For example, of 178 LB in Turkey, 75.30% (*n* = 134) indicated they had perpetrated at least one incidence of restricting, monitoring, and controlling behavior. In Denmark, the rate was 48.70% (*n* = 132, *χ^2^*(1, *n* = 449) = 31.42, *p* < .001, *Φ* = .265). With regard to sexual orientation, we also found significant differences between lesbians and bisexuals for psychological violence measures (except for dominance/intimidation behaviors). For all other types, bisexuals reported inflicting more psychologically abusive acts to their partners compared to lesbians. For instance, out of 205 bisexuals, 65.90% (*n* = 135) indicated perpetrating at least one incidence of restricting, monitoring, and controlling behavior. The rate was 53.70% (*n* = 131 out of 244) for lesbians, *χ^2^*(1, *n* = 449) = 6.83, *p* < .01, *Φ* = .123.

**Table 2.** Frequencies of Psychological Intimate Partner Violence Perpetration Regarding the Country and Sexual Orientation.

	Country			Sexual Orientation		
	Denmark(*N* = 271; *f* = 60.4)	Turkey(*N* = 178; *f* = 39.6)	Chi- square	Lesbians(*N* = 244; *f* = 54.3%)	Bisexuals(*N* = 205; *f* = 45.7)	Chi-square
Restrictive Engulfment	132;48.70%	134;75.30%	*χ*^2^= 31.42*** Φ = .265	131;53.70%	135;65.90%	*χ*^2^= 6.83** Φ = .123
Denigration	90;33.20%	97;48.70%	*χ*^2^= 20.06*** Φ = .211	91;37.30%	96;46.80%	*χ*^2^= 4.17*Φ = .096
Hostile Withdrawal	204;54.40%	171;96.10%	*χ*^2^= 33.74***Φ = .274	197;80.70%	178;86.80%	*χ*^2^= 3.00*Φ = .082
Dominance/Intimidation	63;23.20%	83;56.80%	*χ*^2^= 26.77***Φ = .244	76;31.10%	70;34.10%	*χ*^2^= 46Φ = .032

*Note.* ***p < .001; **p < .01; *p < .05.

### Correlation Analyses

We then explored associations among study variables. Accordingly, we computed zero-order correlations separately for Denmark and Turkey, as summarized in Table 3. The association of internalized heterosexism was significant for all types of psychological violence perpetration variables, for samples from both Denmark and Turkey. LB with higher levels of internalized heterosexism were more inclined to use psychological aggression perpetration toward their partners. Moreover, all types of psychological abuse perpetration were significantly and strongly related. Lesbians and bisexuals’ perpetration of one type of psychological aggression increased the risk of use of different types in both countries.

**Table 3.** Cronbach Alphas, Means, Standard Deviations, and Intercorrelations Among Study Variables.

							Denmark (*N* = 271)			Turkey (*N* = 178)		
Variables	*1*	*2*	*3*	*4*	*5*	*6*	*M*	*SD*	*α*	*M*	*SD*	*α*
1. Sexual orientation	1	.30**	.14*	.16	.02	-.04	-	-	-	-	-	-
2. Internalized heterosexism	.00	1	.27**	.22**	.33**	.13*	109.66	32.37	.91	113.79	32.97	.90
3. Restrictive engulfment	-.09	.24**	1	.33**	.35**	.39**	2.39	4.10	.73	4.24	5.52	.78
4. Denigration	-.10	.27**	.59**	1	.57**	.59**	1.47	3.22	.71	2.55	4.55	.79
5. Hostile withdrawal	-.07	.23**	.50**	.44**	1	.48**	7.15	8.34	.87	12.71	8.33	.84
6. Dominance/Intimidation	-.14	.20**	.67**	.76**	.58**	1	1.21	3.47	.85	2.56	4.79	.83

*Note*. Sexual orientation was coded as lesbian = 1 and bisexual = 2. Correlations above the diagonal represent Denmark and correlations below the diagonal represent Turkey.

*p < .05; **p < .01

### Moderation Analyses

We next performed four moderation analyses to understand the moderating roles of sexual orientation and country on the relationship between internalized heterosexism and each type of psychological violence perpetration (restrictive engulfment, denigration, hostile withdrawal, and dominance/intimidation). This was achieved using PROCESS (Version 3.4, Model 2; please see Figure 1) provided by Hayes (2019). For each of the four separate dependent variables, we reported the direct and conditional effects using 5,000 bootstrap samples.

For restrictive engulfment, the model was significant (*R^2^ = .*10, *F* (5, 443) = 9.95, *p* < .001) as shown in Table 4. The direct association between internalized heterosexism and restrictive engulfment was significant: *β* = .038, 95% CI [.025, .051]. People with higher internalized heterosexism tended to exert more controlling behaviors toward their partners. The direct effect of the country on restrictive engulfment perpetration was also significant: *β* = 1.659, 95% CI [.762, 2.556]. People in Turkey were more prone to use controlling behaviors. We found no conditional effects of sexual orientation and country.

For denigration, the model was significant: *R^2^ =* .09*, F* (5, 443) = 8.35, *p* < .001 as shown in Table 4. The direct association between internalized heterosexism and denigration was significant: *β* = .030, 95% CI [.019, .041]. People with higher internalized heterosexism were more likely to be verbally abusive towards their partners. The direct effect of the country on denigration was also significant: *β* = .958, 95% CI [.234, 1.682]. People in Turkey were at a higher risk of use of verbal abuse. The conditional effects of sexual orientation and country were not significant.

**Table 4.** The Summary of Moderation Analyses.

	*β*	*SE*	Boot LLCI	Boot ULCI
Dependent—Restrictive Engulfment				
Internalized heterosexism	.038	.007	.025	.051
Sexual orientation	−.124	.455	−1.017	.770
Internalized heterosexism × Sexual orientation	−.013	.014	−.040	.014
Country	1.659	.456	.762	2.556
Internalized heterosexism × Country	.007	.014	−.020	.033
*R^2^ =* .101, *F* (5, 443) = 9.949, *p* = 000				
Dependent—Denigration				
Internalized heterosexism	.030	.005	.019	.041
Sexual orientation	−.417	.367	−1.139	.305
Internalized heterosexism × Sexual orientation	−.016	.011	−.037	.006
Country	.958	.368	.234	1.682
Internalized heterosexism × Country	.015	.011	−.007	.036
*R^2^ =* .086, *F* (5, 443) = 8.354, *p* = 000				
Dependent—Hostile Withdrawal				
Internalized heterosexism	.077	.012	.054	.101
Sexual orientation	−1.314	.794	−2.874	.245
Internalized heterosexism × Sexual orientation	.010	.024	-.037	.057
Country	5.579	.796	4.014	7.144
Sexual orientation × Country	−.034	.024	−.081	.013
*R^2^ =* .180, *F* (5, 443) = 19.497, *p* = 000				
Dependent—Dominance/Intimidation				
Internalized heterosexism	.024	.006	.013	.036
Sexual orientation	−.095	.394	−.1.679	−.131
Internalized heterosexism × Sexual orientation	−.026	.012	−.049	−.002
Country	1.309	.395	.532	2.085
Internalized heterosexism × Country	.013	.012	−.010	.036
*R^2^* = .077, *F* (5, 443) = 7.242, *p* = 000				

*Note.* 10000 bootstrap samples. LLCI: lower level of confidence interval; ULCI: upper level of confidence interval.

We obtained the same results for hostile withdrawal as for restrictive engulfment and denigration (Table 4). The direct associations between (a) internalized heterosexism and denigration, *β* = .077, 95% CI [.054, .101] and (b) country and denigration, *β* = 5.579, 95% CI [4.014, .7.144] were significant. No conditional effects of sexual orientation and country were noticed.

For dominance/intimidation, we observed a different pattern (Table 4). As with restrictive engulfment, denigration, and hostile withdrawal, the model was significant: *R^2^ =* .08*, F* (5, 443) = 7.24, *p* < .001. In the model, all the direct effects on dominance/intimidation by internalized heterosexism, *β* = .024, 95% CI [.013, .036], sexual orientation, *β* = -.095, 95% CI [−1.679, −.131], and country, *β* = 1.309, 95% CI [.532, .2.085] were significant. The conditional effect of sexual orientation between internalized heterosexism and dominance/intimidation was also found to be significant: *β* = −026, 95% CI [−.049, −.002]. The interaction term accounted for a significant proportion of the variance in dominance/intimidations, *∇R^2^* = .01, *∇F*(1, 443) = 4.395, *p* < .05, after the direct effects were controlled. Examination of the interaction showed that lesbians (but not bisexuals) with increased internalized heterosexism perpetrated more dominance/intimidation-related behaviors. The interaction effect was true for both Denmark: *β* = .031, 95% CI [.012, .050], *t*(443) = 3.24, *p* < .001; and Turkey: *β* = .044, 95% CI [.022, .066], *t*(443) = 3.89, *p* < .001. In other words, the conditional effect of country between internalized heterosexism and dominance/intimidation appeared to be nonsignificant, *β* = .013, 95% CI [−.010, .036].

## Discussion

The aim of this study was first to examine the frequency of psychological IPV perpetration with a focus on potential differences according to sexual orientation and country. Then, we investigated the moderating roles of sexual orientation and country on the association between internalized heterosexism and the use of psychological IPV.

The findings together imply that psychological IPV perpetration is frequent among LB in both countries, which implies that psychological IPV perpetration is a concern that needs to be addressed, rather than the common belief that IPV is a heterosexual and male perpetrated experience. The symptoms of emotional violence (such as diminished self-esteem) might be more challenging to overcome than other forms of violence; thus, this could lead to a higher rate of retrospective reporting (Head & Milton, 2014) in general. Furthermore, it might be more intense and hurtful for LB women because of the effect of additional stress factors. Moreover, the reciprocal feature of psychological violence (Follingstad & Edmundson, 2010) may also explain the high rates in both Turkey and Denmark.

We found a higher prevalence of IPV perpetration in Turkey than in Denmark. The increased vulnerability of the LGBTQ+ population in Turkey and the lack of protection from discrimination (as demonstrated by the ILGA rating) can be conceptualized as the objective distress described in the minority stress model. In light of this, reduced internalized heterosexism and prevalence of IPV perpetration in Denmark could indicate that less objective discrimination and stressful events (as a result of a longer history of LGBTQ+ antidiscrimination efforts) and legal and institutional recognition result in less internalized heterosexism and consequently less IPV perpetration amongst LB women. Besides, if legal recognition leads to improved trust in authorities, this could lower the threshold for reporting IPV to governmental institutions. This might explain the lower rate of IPV perpetration in Denmark.

From a minority stress perspective, we know that individuals living in tolerant societies are allowed to have more complex identities integrated with other aspects of people’s life. Complex identities increase their resilience toward minority stress (Meyer, 2003). The present study indicates that LB in Turkey report more IPV perpetration toward their partners than LB in Denmark. Perhaps the higher level of IPV perpetration reflects that LB in Turkey live in a country that, under a whole, is more hostile toward sexual minorities. Hence, the current study suggests that one consequence of hostile attitudes toward sexual minorities could be increased levels of internalized heterosexism, resulting in IPV perpetration toward one’s partner. Furthermore, one can also hypothesize that increased levels of IPV perpetration are both a symptom of internalized heterosexism and also a factor that further contributes to it. Thus, the present study adds to the empirical evidence supporting the minority stress model. The findings should encourage the further improvement of antidiscrimination measurements.

Conversely, from an intersectional perspective, higher internalized heterosexism (and thus psychological IPV perpetration) could also be the result of living in a collectivist culture, where the central importance of the family can serve to reproduce a heterosexist ideology in society. According to the intersectional perspective in psychological research, we should be open to different causal mechanisms between social groups. Future research should explore the role played by culture (i.e., collectivism) as a mechanism that mediates the relationship between internalized homophobia and IPV in different contexts. Indeed, in a recent study conducted in Turkey, Toplu-Demirtaş et al. (2020) found that college students who valued hierarchy and distinctive roles in the community (i.e., vertical collectivism) tended to be more supportive of male dominance and gender differentiation, which in turn led to greater acceptance of IPV myths, which in turn could be used to justify dating violence towards dating partners. Nonetheless, the effect of collectivist culture on IPV in heterosexual relationships (Leisring, 2013) is likely different than the effect it has on IPV in LB’s relationships. In Turkey, collectivist culture, which values family over individual wants and desires, intersect with other stressors, that is, sexism, heterosexism, and intense social, legal, and political discrimination. Thus, collectivist culture may add to the minority stress experienced by LB in Turkey, leading to a higher prevalence of IPV perpetration. This is in contrast with LB in Denmark, who live in a dominant individualistic culture which encourages following individual desires, such as defining your own sexuality.

The different societal contexts and policies regarding LGBTQ+ rights in both Denmark and Turkey may affect the prevalence rates of psychological violence perpetration. For instance, the lack of recognition (social validation) as LB and support to LB may lead to isolation from society and greater attachment to their partners (West, 2002). Thus, the perpetrator’s dependency and her partner’s desire to be independent can create a risk for partner violence (Renzetti, 1988). Moreover, in Turkey, the sense of independence and autonomy may be perceived as a threat within a romantic relationship in a country with more collectivist tendencies (e.g., placing a high value on relationship harmony and intimacy) compared to Danish society. Here, LB in Turkey are more likely to create a sense of “fusion” in their relationship than LB in Denmark. High rates of IPV perpetration in Turkey may also signal the issue of reciprocity regarding psychological violence (Follingstad & Edmundson, 2010), although the idea of “the mutual battering” in lesbian/bisexual relationships is controversial (Peterman & Dixon, 2003). Furthermore, the measurement tool we used does not assess the intentions behind those behaviors. Thus, it is limited in its ability to determine whether behaviors may have been used in self-defense or some other protective function within the relationship (Murray & Graves, 2012).

Finally, it is noteworthy that even in Denmark, one of the highest-rated countries for LGBTQ+ rights, minority stress (in the form of internalized heterosexism) is still common. This suggests that institutional and legal reforms aimed to improve the objective distress according to the minority stress model (Meyer, 2003) may not be sufficient enough to ameliorate internalized heterosexism.

Regardless of country, some differences between LB also emerged. Bisexuals (65.9%) compared to lesbians (53.7%) used more controlling behaviors, which were intended to limit and control the partner’s activities and social contacts via jealousy and possessiveness to increase partner dependency. Similarly, bisexuals (46.8%) perpetrated more verbal attacks than lesbians (37.3%), which were intended to humiliate and degrade the partner’s self-esteem and self-worth. Compared to other studies (Balsam & Szymanski, 2005), our findings were in line with much of the literature. It may be the experience of marginalization, which is a unique stress for bisexuals both from the LGBTQ+ and heterosexual communities (Guidry, 1999), which leads bisexual women to be in more conflict in their romantic relationships. Moreover, biphobia may lead bisexuals to feel isolated and distant from others, which can prevent them from noticing and defining their violent behavior. This could be a risk for the normalization and perpetuation of the violence.

Heteronormativity and heterosexism can lead to the perpetration of psychological IPV (Balsam, 2001; Tigert, 2001) among LB’ romantic relationships. For instance, Tigert (2001) argues that the perpetration of violence might be a response to cultural oppression and traumatization. LB women in Turkey are more oppressed than those in Denmark (ILGA-Europe, 2019), which could affect the prevalence rates of psychological violence in Turkey.

We also considered the correlations among study variables noteworthy, which both replicated results of prior studies and offered novel findings. In both countries, LB with higher internalized heterosexism reported more perpetration of each type of psychological IPV, consistent with results in the literature (Tigert, 2001). Experiencing daily social and institutional structures of heterosexism can be internalized, affecting the self-concept. In particular, feeling shame about sexual orientation may be reflected by violent behavior. The correlations also documented strong associations between restrictive engulfment, denigration, hostile withdrawal, and dominance/intimidation perpetration among LB as in the heterosexual community (Toplu-Demirtaş, 2015).

Turning to our hypotheses regarding moderation, we found significant direct and conditional effects. We confirmed our third hypothesis that the participants in our study with higher internalized heterosexism tended to commit more psychological IPV, regardless of the type, which mirrors findings in the literature (as discussed above). Regarding conditional effects, our results revealed no moderation of country on the association between internalized heterosexism and psychological IPV perpetration, in contrast to what we predicted. At this point, we should point out that the influence of subtle or overt heteronormativity can have an impact on violent behavior among LB towards LGBs who live in different social contexts.

Regarding the conditional effect of sexual orientation on the association between internalized heterosexism and psychological IPV perpetration, our hypothesis was partly supported. There is a moderation effect of sexual orientation only for dominance/intimidation. Lesbians (in both countries) with higher internalized heterosexism committed more dominance-related psychological IPV. However, we are not able to discuss this finding at this stage because of the lack of existing literature. To determine a conclusion, we suggest that researchers continue to conduct studies on the link between internalized heterosexism and psychological IPV perpetration with a focus on the multidimensionality of psychological violence and sexual orientation. Dominance was the type of psychological aggression most strongly correlated with physical abuse among heterosexuals (Toplu-Demirtaş et al., 2018) and LB (Chong et al., 2013). This finding suggests that dominance-related psychological violence may be a particularly significant risk factor for further physical abuse among lesbians in both countries.

### Limitations

Despite the strengths of the findings, there were several limitations with the results of the current study, and these should be taken into consideration when interpreting the results. First, the data were based on self-reporting. Second, our participants from both countries were predominantly students and/or employed, young, educated, and nonreligious lesbian and bisexual women. Thus, the results might be different for LB with different demographic backgrounds. We know from other studies on minority stress that higher social class background and education are protective factors towards the potential harmful effects of social stigma (Meyer, 2003). From an intersectional perspective, it could therefore be that the detrimental effects of stigma are stronger among LB from lower social classes without access to higher education or employment, both in Turkey and Denmark. Moreover, we collected online data with the help of announcements from LGBTQ+ organizations on their social media accounts, which means that the recruitment process targeted lesbian and bisexual women who were more “out” and active in the community and with access to the Internet. Therefore, our sample may not reflect the views of the larger lesbian and bisexual population. Third, we used the same psychometrically sound instruments for data collection; however, their estimates of validity and reliability were separately established within each culture. Cross-cultural equivalence of the instruments was not the primary focus and thus not assessed in the current study. Lastly, the study is cross-sectional in nature; therefore, causality cannot be inferred.

### Implications for Research and Practice

Our findings have cross-national implications for further research studies. Further research is warranted with different samples of LB from diverse backgrounds in Turkey and Denmark to confirm the results of the current study. In addition, LB individuals who report more aggression toward their partners in Turkey should be further examined in cultures where same-sex relationships have not received acceptance. Future research could also involve queer and trans women to explore any similarities and differences among these groups. Bisexual women in both Turkey and Denmark mostly reported more violent behaviors toward their partners than lesbians. The silence and invisibility surrounding bisexuals may make them more vulnerable and place them at higher risk of mental health issues (Bayer et al., 2017; Kerr et al., 2015). Research on IPV among bisexuals mostly focuses on being a victim of IPV. However, experiencing biphobia in and outside of the community (Guidry, 1999) might further increase the risk of being a perpetrator of IPV. We believe that qualitative research may provide further insight into bisexuals’ violent behavior toward their partners.

We found significant associations between internalized heterosexism and reported IPV perpetration. This could indicate that internalized heterosexism, understood as one type of minority stress among lesbian and bisexual women, might result in IPV towards their partners. This consequence should be explored further and deserves attention among academics and policymakers alike. Moreover, the association between internalized heterosexism and IPV could also shed light on the higher prevalence of mental health problems among the LGB population (Meyer, 2003). It is possible there may be a dynamic relation at play, where IPV perpetration is a moderator of a higher prevalence of mental health challenges as well as its consequence. In the future, the minority stress model could be further developed by implementing the prevalence of IPV perpetration as both a moderator of mental health challenges and an understudied consequence.

Mental health professionals working with LGBTQ+ individuals may benefit from the understanding that internalized heterosexism is associated with LB women’s perpetration of psychological IPV. Furthermore, they should be ready to address the adverse consequences of experiencing IPV and the role this might play in maintaining mental health challenges among LGBTQ+ clients. Mental health professionals should also advocate for social justice, which has—for the last two decades—been regarded as “a professional imperative” (Myers et al., 2002), in order to ameliorate minority stress affecting gender and sexual minorities. Thus, these results indicate that public recognition and legal measures aimed to protect sexual minorities could affect the prevalence of IPV perpetration and improve the livelihood of LB women and other members of the LGBTQ+ community. Policymakers should, therefore, embrace a proactive antidiscrimination strategy, such as legal protection and campaigns against hate-speech, to improve the lives of LGBTQ+ people (ILGA-Europe, 2020). On the flip side, the legitimization of sexual orientation and gender identity seems not to emancipate LGBTQ+ people from IPV. At this point, IPV service providers should be more knowledgeable about the genuineness of LGBTQ+ IPV and use inclusive language and nongendered assumption of the violence to have LGBTQ+ friendly social and psychological services (Ovesen, 2020).

## Concluding Remarks

There are several strengths in the current study. First, there has been no study to date about psychological IPV perpetration among LB in Denmark; therefore, it is the first study to investigate psychological aggression in Denmark. Second, it is the first study to explore psychological aggression among LB in a multidimensional way. Third, the MMEA seems like a promising instrument for measuring psychological aggression among LB in both the English and Turkish languages. Fourth, the current study implies that lesbians and bisexuals might have different psychological violence perpetration experiences. Lastly, this is the first study to examine the moderating roles of sexual orientation country background in the link between internalized heterosexism and psychological IPV perpetration.

## Author's Note

The work presented here was largely performed while the first author was a post-doctoral researcher at Copenhagen University [and is now at Oslo University].

## Note

1. Only one reported to be 17 years old; thus, we decided keep them.

## Author Biographies

**Esra Ummak**, PhD, is a visiting scholar in the department of psychology at Oslo University. They completed their first post doc in Psychology Department at the Université Libre de Bruxelles and second post doc in the Psychology Department at Copenhagen University. Their research focuses on the mental health of queers and queers of color, intimate partner violence and the social cure approach to social identity. Currently, their primary research interest relates to addressing the significant knowledge gap on mental health challenges of LGBTQI+ and ethnic/racial minorities.

**Ezgi Toplu-Demirtaş**, PhD, is an assistant professor in the Department of Psychological Counseling and Guidance at MEF University and was a Fulbright post doctoral researcher in the Department of Family and Child Sciences at Florida State University. Her research focuses on gender, dating violence, controlling behaviors, cyber dating violence, dating infidelity, LGBTI issues, adult attachment, multicultural issues in counseling, and preventive counseling. Currently, her primary research interest relates to bridging the gap between research and practice in the area of dating violence.

**Reidar Schei Jessen**, MSc in clinical psychology, is a PhD candidate at the University of Oslo/Department of Psychology and Oslo University Hospital. His research interests are focused on gender, sexuality, and development of self and identity, with a special emphasis on minority issues.
